# Synchronous human–dolphin foraging increases fishing success despite prey unpredictability

**DOI:** 10.1093/beheco/arag106

**Published:** 2026-08-28

**Authors:** Alexandre M S Machado, Fábio G Daura-Jorge, Damien R Farine, Penny Tarling, Sergio Escalera, Albert Clapés, Mauricio Cantor

**Affiliations:** Departamento de Ecologia e Zoologia, Universidade Federal de Santa Catarina, Campus Universitário Córrego Grande, Florianópolis, SC 88035-972, Brazil; Department of Collective Behaviour, Max Planck Institute of Animal Behaviour, Am Obstberg 1, 78315, Radolfzell, Germany; Departamento de Ecologia e Zoologia, Universidade Federal de Santa Catarina, Campus Universitário Córrego Grande, Florianópolis, SC 88035-972, Brazil; Department of Collective Behaviour, Max Planck Institute of Animal Behaviour, Am Obstberg 1, 78315, Radolfzell, Germany; Department of Evolutionary Biology and Environmental Science, Universität Zürich, Winterthurerstrasse 190, 8057, Zürich, Switzerland; Division of Ecology and Evolution, Research School of Biology, Australian National University, 46 Sullivans Creek Road, Canberra, ACT 2600, Australia; Facultat de Mathemàtiques i Informàtica, Universitat de Barcelona, Gran Via de les Corts Catalanes, 585, 08007 Barcelona, Spain; Facultat de Mathemàtiques i Informàtica, Universitat de Barcelona, Gran Via de les Corts Catalanes, 585, 08007 Barcelona, Spain; Computer Vision Center, Universitat Autònoma de Barcelona, Edifici O, Campus UAB, 08193 Bellaterra (Cerdanyola), Barcelona, Spain; Facultat de Mathemàtiques i Informàtica, Universitat de Barcelona, Gran Via de les Corts Catalanes, 585, 08007 Barcelona, Spain; Computer Vision Center, Universitat Autònoma de Barcelona, Edifici O, Campus UAB, 08193 Bellaterra (Cerdanyola), Barcelona, Spain; Departamento de Ecologia e Zoologia, Universidade Federal de Santa Catarina, Campus Universitário Córrego Grande, Florianópolis, SC 88035-972, Brazil; Department of Fisheries, Wildlife and Conservation Sciences, Marine Mammal Institute, Oregon State University, 2030 SE Marine Science Drive, Newport, OR 97365, United States; Department for the Ecology of Animal Societies, Max Planck Institute of Animal Behaviour, Universitätsstraße 10, Konstanz, D-78464, Germany; Centro de Estudos do Mar, Universidade Federal do Paraná, Av. Beira-mar s/n Pontal do Sul, Pontal do Paraná, PR 93255-970, Brazil

**Keywords:** foraging specialization, habitat condition, predator–prey, prey availability, social foraging, *Tursiops truncatus*

## Abstract

Foraging theory predicts that individuals adjust their behavior in response to environmental conditions and prey availability. One solution to finding and capturing unpredictable prey is social foraging, yet it remains unclear whether its benefits arise from behavioral coordination itself or simply from predators co-occurring under favorable environmental conditions. We examine how environmental conditions shape the outcome of social foraging between predators—dolphins and human fishers—that forage together on patchy, ephemeral migratory mullet schools. Using fisher catch as our empirical measure of foraging success, we test whether mullet abundance and catch are influenced by local environmental conditions, or whether social foraging buffers fishers from fluctuations in prey availability. Using confirmatory path analysis, we investigate the links among environmental conditions, mullet abundance, predators' abundance, and synchronous behavior to determine their relative influence on prey capture. Our results indicate 2 pathways to higher prey capture. First, fishers catch more mullet when these are more abundant (typically under northerly winds), but environmental conditions do not predict dolphin–fisher foraging synchrony. Second—and independently—more dolphins are present during flood tides, which leads to more fishers, more synchronous foraging, and more mullet caught. These moment-by-moment analyses demonstrate that behavioral coordination, rather than prey availability or shared environmental responses alone, is the primary driver of fisher success. More broadly, our results provide a mechanistic understanding of how social foraging can buffer predators against environmental variability, highlighting the role of coordination in overcoming prey unpredictability.

## Teaser

Among wild dolphins that cooperatively forage with artisanal fishers, we show that foraging success depends less on how many fish are present and more on both dolphin and human predators being together and coordinating their actions. Thus, predators can reduce their dependence on cues from environmental conditions and jointly exploit fleeting prey by cooperating, revealing when and why interspecies social foraging pays off. Dolphins and fishers succeed by working together as cooperation outweighs prey abundance.

## Introduction

Animals live in ever-changing environments, and their foraging success depends on matching their foraging effort with the activity of their prey (Miller-Rushing et al. 2010; McNamara et al. 2011). For example, predatory birds can track the variation in prey activity over the course of the day, foraging more when their prey are more active (Lang et al. 2019). The temporal availability of ephemeral prey and/or the spatial location of patchily distributed prey might be predicted by current environmental conditions (eg tidal currents; Embling et al. 2012). For example, Baleen whales forage in areas where environmental conditions predict the abundance of krill patches (Barlow et al. 2020). However, for some species, prey availability and environmental conditions can be disconnected, particularly when environmental drivers operate at broader spatial or temporal scales than those experienced locally by predators. In such cases, the lack of reliable local environmental cues can make foraging more challenging for predators that hunt highly mobile prey (Navarro et al. 2016). One proposed solution to address this challenge is social foraging, whereby predators use social information and coordination to reduce uncertainty about prey location and availability (Giraldeau and Caraco 2000; Egert-Berg et al. 2018). In addition, collective decision-making can improve the detection and tracking of patchy prey because groups can pool imperfect information, averaging individual errors and collectively sensing environmental gradients that may be difficult for single individuals to detect (Kao and Couzin 2014). For example, predators can use social cues to gain information either inadvertently (eg bats can eavesdrop on their conspecifics to discover new food patches; Dechmann et al. 2009) or actively (eg social songbirds call for conspecifics to forage on the same food patch; Brown et al. 1991) about the distribution or availability of their prey (Kohles et al. 2022). For predators that hunt in groups, social foraging can then stimulate even greater collective benefits when multiple individuals cooperate to hunt their common prey (Krebs and Inman 1992), a pattern also observed in seabirds, such as gannets, under variable foraging conditions (Thiebault et al. 2014). However, these benefits may be offset by costs, such as increased competition or interference among predators, meaning that the net benefits of social foraging are not always straightforward (Laguë et al. 2012).

Social foraging is relatively widespread among taxa (Lang and Farine 2017). However, determining whether individuals benefit from foraging in groups or if individuals are simply more likely to form groups when prey are more abundant is often challenging. This is because addressing this question requires understanding how environmental conditions at different spatial scales predict prey availability, how prey availability affects the predators' ability to catch prey, and whether predators catch more prey when hunting together. For example, there are many predator species in which individuals aggregate in specific environments to catch a common and predictable prey (eg brown bears *Ursus arctos* fishing for migrating Pacific salmon; Deacy et al. 2016), but it remains unclear whether they gain any additional benefits by hunting at the same time and place. By contrast, predators in smaller packs (eg killer whales *Orcinus orca* hunting marine mammals; Baird and Dill 1996), or in larger packs (eg African wild dogs *Lycaon pictus* hunting large prey; Fanshawe and Fitzgibbon 1993), can be more likely to catch more or larger prey, although this relationship could also be driven by packs being more likely to form when prey are more abundant. Finally, social associations among predators can change under different prey abundance regimes (eg African lion *Panthera leo* prides fission or fusion according to prey availability; Mbizah et al. 2020), but it is unclear how this translates to the foraging performance of individuals. It remains a challenge to simultaneously track and relate environmental conditions, prey abundance, and predator behavior, making the causal drivers underlying the benefits that predators gain by foraging socially largely unknown.

This limitation makes it difficult to determine whether the benefits of social foraging arise from behavioral coordination or simply from individuals co-occurring under favorable environmental conditions. This is particularly relevant in systems where prey is patchy and ephemeral, as collective foraging may allow predators to overcome spatiotemporal unpredictability in resource availability. Although most work on social foraging focused on conspecific interactions, coordinated foraging across species remains less well understood, particularly when individuals rely on heterospecific cues (Seppänen et al. 2007). We address this challenge here by studying a rare but highly accessible system that allows us to test how predator co-occurrence and behavioral coordination influence foraging success under conditions of high prey unpredictability. Although the processes driving prey availability operate across spatial scales, our study focuses on how these processes manifest as temporal variation at a fixed interaction site. By combining direct estimates of prey availability with environmental and behavioral data, we disentangle the relative roles of prey abundance, predator co-occurrence, and synchronous foraging in shaping foraging outcomes, providing a mechanistic understanding of how social foraging operates in such dynamic environments. Together, this approach allows us to test whether the benefits of social foraging arise from behavioral coordination rather than shared responses to environmental variation, advancing our understanding of collective foraging in dynamic environments.

In southern Brazil, the century-old traditional fishery involving wild bottlenose dolphins (*Tursiops truncatus gephyreus*) and artisanal net-casting human fishers targeting migratory mullet (*Mugil liza*) (Simões-Lopes et al. 1998), who operate from the shoreline using a sit-and-wait tactic and cast their nets in response to dolphin cues, is one of the last cases of human–wildlife cooperation, whereby human and nonhuman animals synchronize their behavior to achieve a potentially mutually beneficial outcome (sensu Cram et al. 2022). The challenge that dolphins face in this system is to catch individual prey in large, fast-moving, and highly coordinated mullet schools (eg Major 1978; Simões-Lopes et al. 1998). By contrast, fishers are highly adept at catching entire mullet schools using their fishing gear, but not when mullet schools are in highly murky estuarine waters where they are hard to track visually and can move beyond the reach of shore-based net-casting fishers (see Cantor et al. 2023), thereby becoming a highly unpredictable prey in space and time (eg Ortega et al. 2020). Recent evidence suggests that these species solve their respective challenges by responding to each other's behavior, ie by foraging synchronously. First, dolphins herd mullet schools toward coastal shallow waters (Cantor et al. 2023) where artisanal net-casting fishers wait for specific cues from the dolphins (stereotyped behaviors, such as sudden back-arching dives or head and tail slaps) that indicate the mullet school location, enabling fishers to cast their nets at the appropriate time and place (Simões-Lopes et al. 1998). Then, dolphins react by remaining underwater and emitting characteristic echolocation terminal buzzes—a clear marker of a foraging bout and suggestive of prey capture (eg Wisniewska et al. 2014)—while foraging on the mullet schools disrupted by, and trapped in, the nets (Cantor et al. 2023). When dolphins and fishers forage in such synchrony, dolphins are almost 3 times more likely to engage in active underwater hunting and fishers are 17 times more likely to catch any mullet compared with independent or nonsynchronous foraging (Cantor et al. 2023), consistent with a mutualistic interaction. Although this previous work has shown that synchronous interactions between dolphins and fishers can enhance foraging behavior and increase the likelihood of successful fishing events, this interpretation assumes that these benefits arise from behavioral coordination itself. However, these studies did not account for variation in prey availability. As a result, it remains unclear whether the observed benefits of synchrony reflect behavioral coordination per se or simple co-occurrence of predators at the interaction site when environmental conditions make prey locally predictable and abundant.

Here, we disentangle the contributions of environmental conditions and prey–predator co-occurrence from those of social foraging in the dolphin–fisher system by combining 3 data streams. First, we sampled the in situ environmental (water, wind, and tide) conditions known to relate to mullet migration (Herbst and Hanazaki 2014; Lemos et al. 2014). Second, we quantified the moment-by-moment abundance of fast-moving mullet schools in the murky waters using a novel deep learning model trained to count mullet in underwater sonar imagery (Tarling et al. 2022). Third, we conducted fine-scale observational tracking of fisher and dolphin abundance, behavior, and the outcomes of their social foraging. We used these data to perform a confirmatory path analysis to test 3 hypotheses. First, is mullet abundance within the reach of net-casting fishers predicted by changes in fine-scale environmental conditions? Mullet movements are shaped by environmental drivers across spatial scales: colder temperatures and southerly winds trigger migration at regional scales, whereas northerly winds bring schools closer to the coast locally (Herbst and Hanazaki 2014; Lemos et al. 2016). Their entry into estuaries—and thus availability at the interaction site—remains less well understood but likely depends on additional fine-scale tidal and hydrodynamic processes (Herbst and Hanazaki 2014; Lemos et al. 2016). We therefore predict that these environmental conditions influence the movements of mullet schools in and out of the estuarine system, thereby affecting local mullet availability where dolphins and fishers forage together. Second, are dolphins and fishers more numerous in moments of high mullet abundance or under specific environmental conditions? We predict that the abundance of foraging dolphins at the interaction site mirrors the periods when mullet schools are more abundant, and that the abundance of fishers in the water, who rely on dolphins as a cue for mullet availability, mirrors the periods of high dolphin abundance. Finally, does the co-occurrence of both predator species result in higher probabilities of successful social foraging? We predict that the incidence of synchronous foraging between dolphins and net-casting fishers correlates with their local abundances and consequently leads to an increased likelihood of mullet being caught by fishers.

## Material and methods

**Figure 1:**
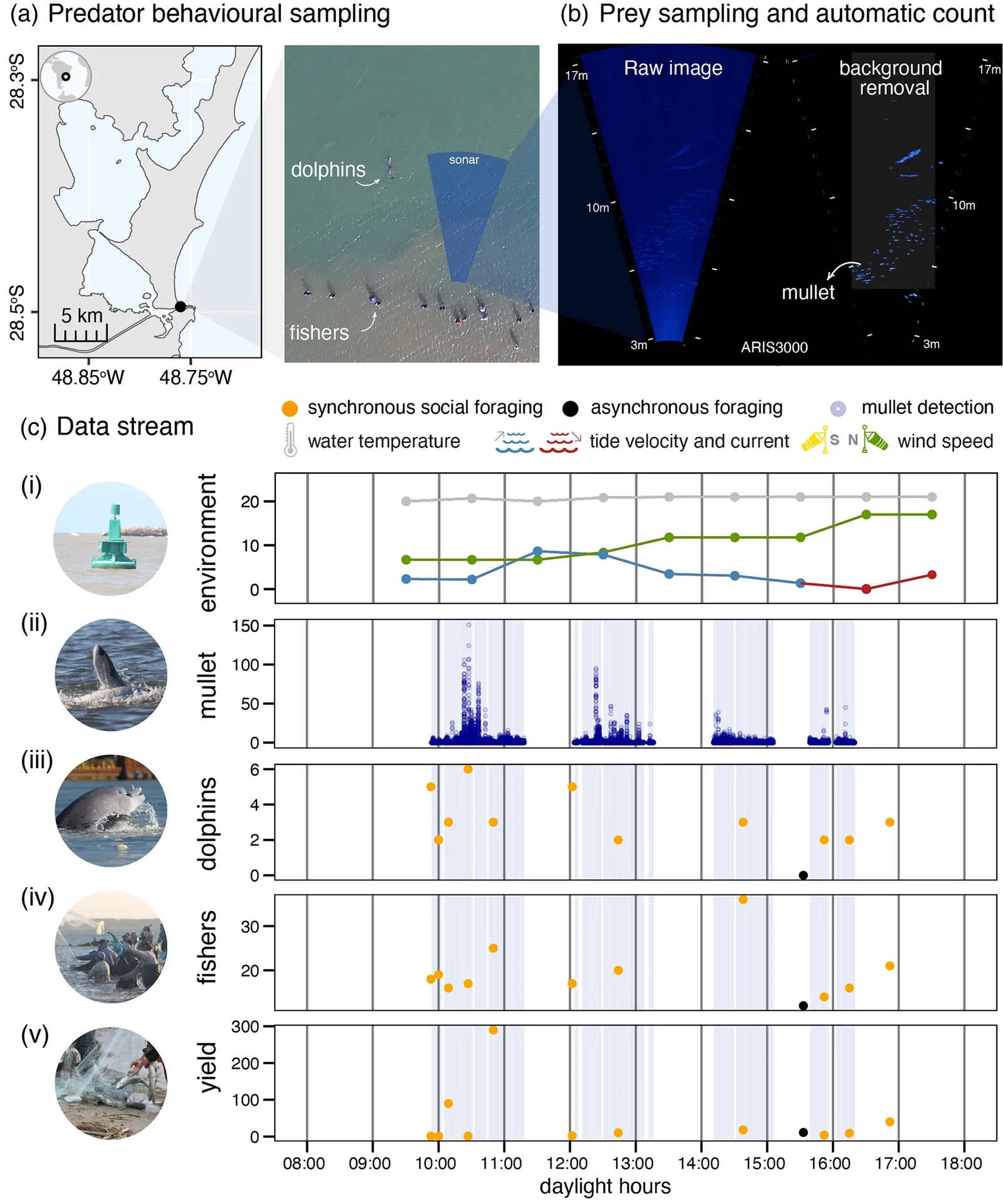
Figure 1. Overview of the study system, sampling methods, and an excerpt of the simultaneously collected and aligned data streams. (a) Sampling of predator and prey co-occurrence and behaviour in the lagoon system of Laguna, southern Brazil. The aerial image shows the predators from above—bottlenose dolphins (centre) and artisanal net-casting fishers (bottom, in line along the edge of the canal)—, and the blue area highlights the underwater image generated by the ARIS3000 sonar sampling, exemplified by (b) the same sonar frame presented on the left as the raw image, and on the right as a processed image with enhanced contrast and removed background. The highlighted rectangular area marks the area cropped to standardize all input samples in the deep learning model with density-based regression used to quantify the mullet abundance. (c) For a sampling day, all environmental and observational data were collected simultaneously with the sonar sampling (shaded blue areas): (i) hourly local environmental conditions: water temperature (°C), wind speed (mph), wind direction (green: northerly; yellow: southerly, absent this day), tide velocity (cm/h), and tidal currents (blue: flood; red: ebb); (ii) mullet school detections are represented by blue dots in the frame-by-frame sonar sampling; the number of (iii) dolphins, (iv) fishers , and (v) mullet caught in synchronous (orange) and asynchronous (black) social foraging events between dolphins and fishers.

We simultaneously collected environmental and behavioral data, both above and underwater, to investigate the temporal variation in dolphin–fisher foraging interactions in Laguna, southern Brazil (Fig. 1). We focused our sampling effort on the primary site where the social foraging between dolphins and fishers takes place: a small beach (ca. 100 m in length) in the inlet canal that connects the estuarine system to the Atlantic Ocean (Cantor et al. 2018). From 9 AM to 5 PM, over 14 d (22 June to 1 July and 4 July to 7 July 2018), we continuously monitored environmental conditions, quantified mullet abundance, and recorded the number and behavior of dolphins and fishers, as well as their foraging interactions (Machado et al. 2026), as detailed below.

### Monitoring environmental conditions

We assessed changes in local environmental conditions to investigate whether they could predict changes in mullet abundance, which could drive temporal variation in the abundance of dolphins and fishers engaged in social foraging and their joint foraging success. Both empirical data and traditional ecological knowledge suggest that the mullet reproductive migration is triggered by a decrease in sea surface temperature and the prevalence of southern winds (Herbst and Hanazaki 2014; Lemos et al. 2016). As mullets migrate northward, a shift from southern to northern winds and an increase in water temperature can bring mullet schools closer to the coast. Tidal currents can also play a role in how mullet schools move along the coast, especially when synchronized with the timing of mullet arrival, as tidal currents can facilitate the movement of schools in and out of estuarine areas (Herbst and Hanazaki 2014; Lemos et al. 2016).

Based on this knowledge, we continuously recorded the water temperature at the interaction site using the sensor from ARIS3000 (see below). At the beginning of each sampling hour, we measured wind speed and wind direction in situ using an anemometer (Instrutherm AD-250). We then classified wind direction as the southern quadrant (originating from the S–SW and S–SE, moving northward) and the northern quadrant (from N–NW and N–NE, moving southward). To assess changes in the tide levels, we obtained data from a weather station located inside the lagoon system (28° 29.1'S, 48° 46.9′W) with a digital water level sensor (Thalimedes OTT Messtechnik GmbH & Co. KG), positioned at 1 m from the bottom (Epagri 2020), and used the hourly tide height measurements to classify tidal currents in each sampling hour as “ebb” or “flood” and to estimate tide velocity as the difference in tide height (in centimeters) from hour to hour.

### Quantifying fine-scale mullet abundance

The high turbidity and low visibility (0.25 to 1.0 m) of the murky waters of the canal pose a major challenge for accurately estimating prey availability. To overcome this challenge, we deployed a high-resolution underwater imaging system—the Adaptive Resolution Imaging Sonar ARIS Explorer 3000 (©Sound Metrics Corp, WA, USA)—that can detect and measure fish under zero-visibility conditions (Cook et al. 2019; Lankowicz et al. 2020; Tarling et al. 2022). The ARIS Explorer 3000 uses 128 beams to emit sound pulses of very high frequencies (up to 3 MHz), converting the returning echoes into digital images. With a sampling rate of around 3 frames per second, the sonar camera generates a bird’s-eye view of a 30° wedge ranging from 2 to 20 m from the sonar (Fig. 1b; Tarling et al. 2022). We mounted the sonar camera on a platform at the edge of the canal slope where the fishers stand, at around 1 m depth, and within the main cooperative foraging hotspot (in the middle of the 100 m wide interaction site; Fig. 1a) to sample the area where dolphins and fishers encounter and capture prey (see Cantor et al. 2023). We manually adjusted the sonar camera position and angle below the water surface to detect mullets from 2 to 20 m from the edge of the slope into the canal. To avoid any disturbance to the dolphins' behavior, we operated the sonar at very high frequencies of 1.8 to 3 MHz, which were 10 to 60 times above the dolphins' audible range (upper-frequency limit of functional hearing: 50 to 169 kHz; Finneran et al. 2008; Houser et al. 2008).

To process the sonar images, we first used the native ARIS Scope software to increase contrast and apply background subtraction, which removes any background noise (ie any static part of the footage), thereby leaving only the mullet in motion (Tarling et al. 2022). This procedure was applied consistently across all recordings and is therefore unlikely to introduce systematic bias in prey detection. We can safely assume that the fast-moving, densely packed schools in the sonar images are mullets because of the size of individual fish, and because this is the dominant schooling fish species in the area from May to July (Simões-Lopes et al. 1998). Given the varying range of the sonar beam across samples, we cropped all analyzed sonar images to a range of 8 to 17 m to avoid any noise from the sea bottom near the edge of the canal (too close to the sonar) and to minimize any bias in mullet abundance due to the maximum sampling area (too far from the sonar). We then exported these video samples and cropped their frames to a standard sampling area of 4 × 8.5 m (Fig. 1b).

We employed our novel density-based deep learning model (Tarling et al. 2022) to accurately count mullet in the sonar images. This deep learning model was specifically developed to process and accurately count dense, fast-moving mullet schools on a moment-by-moment basis from the low-resolution sonar images (Tarling et al. 2022) by using a density-based regression approach derived from methods commonly used to count people in crowds (Liu et al. 2018; Oh et al. 2020). Therefore, mullet abundance in each sonar frame is inferred from spatial density patterns and is robust to partial observations within the sampling window. To address the lack of labeled biological population data, the model was simultaneously trained on a self-supervised task to learn the relative abundance of fish in pairs of overlapping image crops from a set of unlabeled images from our ARIS sonar sampling. To ensure the model was well adapted to varied and imbalanced datasets of images captured in the wild, which often contain noise (eg nets and dolphins) and occlusions, we also incorporated uncertainty regularization (Tarling et al. 2022).

Finally, to assess prey patchiness, we tested if the mullet school sizes in the sonar imagery followed a power law distribution. We estimated school sizes by considering the total mullet counts frame-by-frame of the sonar imaging (Figs. S1 to S3). Because the sonar samples a restricted acoustic volume, the prey quantified within the sonar field of view may not always represent the total size of mullet schools. However, as schools moved through the beam, these estimates captured the fine-scale temporal variation in mullet availability that is relevant to the predators' foraging decisions.

### Quantifying predator abundance, behavior, and social foraging

We used behavioral all-occurrence sampling (Altmann 1974) to record every instance of 2 specific, predefined behaviors: fisher foraging (net casts) and dolphin cues (ie a stereotyped behavior, such as a sudden dive arching their back or head and tail slaps; see Simões-Lopes et al. 1998). Because both predators regularly arrived at, and departed from, the interaction site, we also recorded at every cast or cue the relative abundance of fishers (numbers of active individuals in the water) and dolphins (number of individuals foraging at the site). We defined a foraging event as the moment when fishers cast their nets; in the context of the dolphin–fisher system, these events can be categorized as “synchronous” or “asynchronous” foraging. Synchronous events characterize the cooperative foraging between humans and dolphins (Cantor et al. 2023) and are defined as when fishers cast nets following a dolphin cue (see Simões-Lopes et al. 1998) that discloses the presence and location of the fish schools and typically increases their joint foraging success (Cantor et al. 2023). By contrast, asynchronous foraging events were defined as instances in which fishers cast their nets either in the absence of dolphins, in the absence of a dolphin cue, or without appropriately following a dolphin cue (ie casting too late and too far from the cue). These cases together represent failures to achieve interspecific coordination. Because all 3 types of asynchronous events typically yield similarly low joint dolphin–fisher foraging success relative to synchronous events (Cantor et al. 2023), we considered them together to contrast coordinated versus uncoordinated foraging states in subsequent analyses.

At each foraging event, synchronous or asynchronous, we counted the number of individual dolphins at the interaction site (within 50 m from the sandbank to the middle of the canal; Fig. 1a), the number of fishers in the water, and the number of fishers that cast a net in response to behavioral cues from dolphins. We limited our counts of dolphins to those that remained present and foraging prior to and after the net-casting events, disregarding nonforaging dolphins that were clearly just passing through the canal. Because our long-term photo-identification data demonstrate that not all dolphins of this small resident population engage in the cooperative fishery (Daura-Jorge et al. 2013; Bezamat et al. 2019; Cantor et al. 2023), we also photo-identified all individual dolphins at the interaction site to discount those known to never interact with artisanal fishers. To assess the outcome of each (either synchronous or asynchronous) foraging event, we inspected all nets cast when fishers returned to the beach and counted the number of mullet caught by fishers, if any.

### Statistical analyses

We used path analysis with local estimation (Lefcheck 2016) to investigate the temporal variation in the rates and outcomes of social foraging between dolphins and fishers. Path analysis can simultaneously test multiple causal hypotheses within a single network, in which 1 variable can act both as a response variable on one path and as a predictor on another path (Shipley 2000). Here, we used a single path model based on the fitting of 5 individual linear mixed models to test the following 5 hypotheses derived from our 3 main hypotheses outlined in the introduction: (i) changes in local environmental conditions affect mullet abundance at the interaction site; (ii) high numbers of dolphins at the interaction site coincide with moments of high mullet abundance, or with specific environmental conditions that are reflective of high mullet abundance; (iii) a high number of fishers in the interaction site coincides with moments of high dolphin abundance, or specific environmental conditions; (iv) synchronous social foraging events between dolphins and fishers are more frequent when both predators are more abundant at the interaction site; and (v) both synchronous social foraging and mullet abundance play a role in defining the outcome (in terms of mullet caught) of the dolphins’ and fishers' joint foraging effort, as reflected in fisher catch. These component models collectively allow us to evaluate the relative contributions of environmental conditions, prey availability, predator co-occurrence, and behavioral synchrony to foraging success.

To perform this analysis, we first selected the observational data (ie counts of dolphins, fishers, foraging events, and mullet caught) that intersected with the sampling time of the sonar data (Fig. 1c). Second, we filtered out sonar frames containing noise (detected objects other than mullet schools, such as nets) and summarized all variables to the respective sampling hour. This filtering affected only a small proportion of the data and was not associated with specific environmental conditions, making it unlikely to bias the results. To quantify mullet abundance for each sampling hour, we summed the number of detections from our deep learning models and divided this total by the number of frames within that hour to account for differences in sampling time. Similarly, to quantify the abundance of dolphins and fishers for each sampling hour, we calculated the mean number of individual dolphins and fishers identified at the interaction site within each hour. Third, we transformed some variables to meet the model assumptions and avoid convergence issues—specifically, the categorical variables tidal currents and wind directions were each transformed into numerical predictors by assigning negative values (−0.5) to ebb currents and to southern winds, and positive values (+0.5) to flood currents and to northern winds. All response variables were transformed using the natural logarithm of 1 plus the input vector because we hypothesized a positive relationship between the nodes of the path model. Although mullet counts at the frame level are skewed, aggregation at the hourly level and log transformation reduced skewness, supporting the use of Gaussian error structures, as confirmed by residual diagnostics (Figs. S5 to S9). Finally, before fitting the path model, we conducted an exploratory data analysis (Zuur et al. 2010) and removed from the analysis 1 sampling hour considered to be an outlier in mullet abundance (ie an unusually high detection that could represent a processing error).

We fitted the path model using linear mixed-effect regression models. For each component model, we included a random intercept for the sampling day to account for day-to-day variation in large-scale factors influencing the mullet migration and an autoregressive correlation structure (AR1) to account for the correlation among sampling hours. We used Fisher's C statistic to assess the overall goodness-of-fit of the single path model and test whether our hypothesized pathways aligned with the relationships indicated by the data. We opted to omit links that were not hypothesized a priori. To check the assumptions of normality and heteroscedasticity, we visually inspected the residuals of each linear mixed model (Figs. S5 to S9). We fitted linear mixed models and the path model using the *nlme* (Pinheiro et al. 2024) and the *piecewiseSEM* (Lefcheck 2016) R packages, respectively. To assess each model fit, we used the theoretical marginal and conditional $R_{GLMM}^{2}$ (Nakagawa et al. 2017).

## Results

We collected data on local environmental conditions, prey abundance, and fisher and dolphin abundance, dolphin cues and net casts, and the number of mullet caught by fishers over 84 h, spanning 14 d. From these data, we processed a total of 550,805 unique frames from the sonar videos, of which 502,939 were free of noise, and 108,619 contained mullet detections (median = 3 mullet, mean ± SD = 9.20 ± 22.36, range = 1 to 697). We found that mullet school sizes followed a power law distribution (Fig. S4), suggesting that the prey was patchily distributed (only a few frames with large schools).

Aligning the sonar and observational datasets resulted in 511 observation events with precise numbers of fishers and dolphins involved. We recorded 280 synchronous social foraging events (when fishers cast nets in response to the dolphins' foraging cue) and 220 asynchronous foraging events, including 133 cases in which fishers cast without or not following a dolphin cue, and 87 cases in the absence of dolphins. There were an additional 7 events in which fishers did not react to the dolphin cues and 4 events in which dolphins were foraging at the interaction site and there were no fishers in the water. In foraging events without dolphins, the number of active fishers in the water ranged from 1 to 31 (median = 7, mean ± SD = 8.99 ± 7.64). Across all foraging events with dolphins present at the interaction site, the number of active fishers in the water ranged from 1 to 36 (median = 22, mean ± SD = 21.06 ± 7.47). Across all foraging events, a total of 1,921 mullets were caught by the fishers, ca. 86% (1,658) of which were caught as a result of synchronous social foraging events.

**Figure 2:**
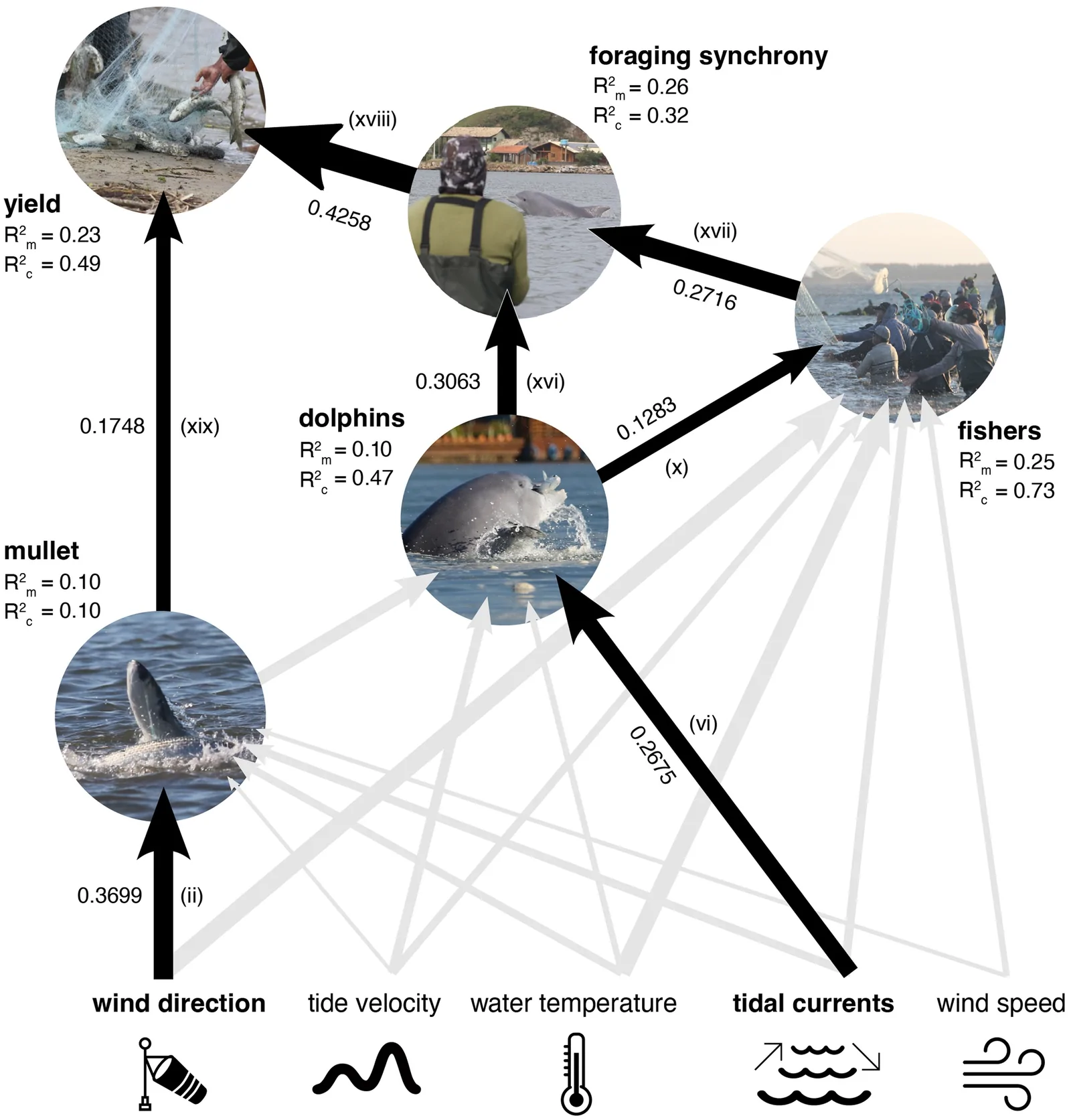
Figure 2. Confirmatory path analysis revealed two non-exclusive pathways that maximize successful foraging outcomes for fishers. The first pathway occurs when dolphins are more abundant, (vi) typically during the flood current of the tidal cycle, (x) which leads to a greater number of fishers and, subsequently, to (xvii) a greater number of synchronous foraging events (xviii), resulting in more mullet caught. In the second pathway, higher mullet catch (xix) is associated with greater mullet abundance, typically during northerly winds (ii). Nodes represent the abundance of prey (‘mullet’) and predators (‘dolphins’, ‘fishers’); whether social foraging events between dolphins and fishers are synchronous (‘foraging synchrony’); and the number of mullet caught by fishers (‘yield’). Paths represent the hypothesised relationships, where black arrows represent statistically supported positive effects (Roman numerals) and paths in grey represent non-significant relationships. Path thickness reflects the relative effect sizes based on standardised path coefficients (given by numbers). See Table 1 for all hypothesised paths and standardised path coefficients. Marginal $R_{GLMM}^{2}$ and Conditional $R_{GLMM}^{2}$ values are given for each model to represent the proportion of the total variance explained by fixed, and both fixed and random effects, respectively.

The path model showed a good fit to the data (C = 44.453; df = 32; *P* = 0.071), indicating that the model adequately fits the data and is consistent with several of the hypothesized relationships among environmental conditions, prey availability, predator behavior, and foraging success in the dolphin–fisher system (Fig. 2). Below, we report the results in relation to the 3 hypotheses outlined in the introduction. Consistent with our first hypothesis, mullet abundance was partially influenced by environmental conditions. Specifically, northerly winds had a clear positive effect on mullet abundance (Table 1, path ii), while changes in local conditions of water temperature and tide had no effect on predicting mullet abundance at the fine spatiotemporal scale of the dolphin–fisher interaction, either within a day (marginal $R_{GLMM}^{2}$ = 0.1) or across days (conditional $R_{GLMM}^{2}$ = 0.1; Table 2). Contrary to our second hypothesis, dolphin abundance was not significantly related to mullet abundance. Instead, the flood phase of tidal currents had a direct and positive effect on the number of dolphins present (Table 1, path vii). Similarly, the number of fishers in the water was not significantly influenced by local environmental conditions. However, there were more fishers present when more dolphins were at the interaction site (Table 1, path x), indicating that fisher presence was more closely associated with dolphin presence than with environmental conditions or prey availability. Supporting our third hypothesis, the co-occurrence of dolphins and fishers increased the likelihood of synchronous social foraging events. Specifically, higher numbers of dolphins (Table 1, path xvi) and fishers (Table 1, path xvii) were associated with more frequent synchronous foraging. In turn, synchronous foraging had a strong positive effect on fisher yield (Table 1, path xviii), which was 2.44 times greater than the effect of mullet abundance itself (Table 1, path xix). Together, these results indicate that behavioral coordination plays a stronger role in determining foraging success than local prey availability. The coefficients of statistically supported paths are highlighted in Fig. 2, and full model results are provided in Table 1 and Tables S1 to S5 and Figs. S5 to S9).

**Table 1:** Path analysis coefficients.

Path	Response	Predictor	Estimate	Std. error	df	Critical value	*p*-value	Std. estimate
i	Mullet abundance	Tide velocity	−0.0389	0.0466	64	−0.8359	0.4063	−0.1000
ii	Mullet abundance	Wind direction	0.6140	0.3037	64	2.0215	0.0474*	0.3699
iii	Mullet abundance	Tidal currents	−0.0054	0.2097	64	−0.0259	0.9794	−0.0035
iv	Mullet abundance	Wind speed	−0.0077	0.0172	64	−0.4501	0.6542	−0.0676
V	Mullet abundance	Water temperature	0.0077	0.1058	64	0.0728	0.9422	0.0128
vi	Dolphins	Tide velocity	0.0257	0.0252	65	1.0188	0.3121	0.1020
vii	Dolphins	Tidal currents	0.2699	0.1112	65	2.4271	0.0180*	0.2675
viii	Dolphins	Water temperature	−0.0059	0.0605	65	−0.0980	0.9222	−0.0152
ix	Dolphins	Mullet abundance	0.0585	0.0642	65	0.9119	0.3652	0.0904
x	Fishers	Dolphins	0.1828	0.0865	63	2.1135	0.0385*	0.1283
xi	Fishers	Tide velocity	−0.0031	0.0210	63	−0.1474	0.8833	−0.0086
xii	Fishers	Wind direction	0.4724	0.2871	63	1.6453	0.1049	0.3084
xiii	Fishers	Tidal currents	0.0492	0.1061	63	0.4637	0.6444	0.0342
xiv	Fishers	Wind speed	0.0068	0.0102	63	0.6679	0.5066	0.0643
xv	Fishers	Water temperature	0.1330	0.0757	63	1.7571	0.0838	0.2396
xvi	Foraging synchrony	Dolphins	0.5001	0.1819	67	2.7494	0.0077**	0.3063
xvii	Foraging synchrony	Fishers	0.3113	0.1392	67	2.2364	0.0287*	0.2716
xviii	Yield	Foraging synchrony	0.8953	0.1961	67	4.5661	0.0000***	0.4258
xix	Yield	Mullet abundance	0.3887	0.1836	67	2.1167	0.0380*	0.1748

Estimates are given for each path between response and predictor variables (see Fig. 2), along with their standard error (std. error), degrees of freedom (df), critical value (the computed test statistic to assess significance of each path), *p*-values (*p < 0.05; **p < 0.01; ***p < 0.001), and standardized coefficient (std. estimate). Standardized coefficients are based on the standard deviation (SD), given that if the predictor goes up by 1 SD, the response goes up by the coefficient value in SD. Therefore, std. estimate can be used to compare the strength of direct and indirect effects.

**Table 2:** Marginal and conditional $R_{GLMM}^{2}$ are given for all response variables of each component linear mixed model of the path analysis.

Response	Family	Link	$R_{GLMM}^{2}$ marginal	$R_{GLMM}^{2}$ conditional
Mullet abundance	Gaussian	identity	0.1	0.1
Dolphins	Gaussian	identity	0.10	0.47
Fishers	Gaussian	identity	0.25	0.73
Synchronous social foraging	Gaussian	identity	0.26	0.32
Yield	Gaussian	identity	0.23	0.49

Marginal $R_{GLMM}^{2}$ represent the variance explained by the fixed effects, while the conditional $R_{GLMM}^{2}$ takes the random effects into consideration.

## Discussion

Our findings address the 3 hypotheses outlined in the introduction and show that synchronous social foraging between apex predators—here, human fishers and wild dolphins—can buffer fisher prey capture against the challenges of exploiting patchy and ephemeral prey, such as migratory mullet schools. Although higher prey abundance—which varies independently of most local environmental conditions—corresponds to greater foraging success (ie number of mullet caught by fishers), this effect is substantially smaller than that of synchronous social foraging between fishers and dolphins. This strong effect of social foraging on fishers' prey capture is consistent with previous interpretations of this system as mutualistic (Simões-Lopes et al. 1998) and further emphasizes the key role of behavioral synchrony between humans and dolphins in facilitating successful fishing events (Cantor et al. 2023). Importantly, however, our empirical measure of foraging success is limited to fisher catch, and we did not directly quantify prey capture by dolphins; therefore, any inference regarding benefits to dolphins remains indirect. More broadly, these results provide a mechanistic explanation for how behavioral coordination can buffer predators against environmental unpredictability, helping to disentangle the effects of coordination from those of predator co-occurrence. This contrasts with classical expectations from functional response models, which typically assume that prey abundance is the primary driver of predation rates, and instead highlights the dominant role of behavioral coordination.

There are multiple routes through which individuals can adapt their foraging behavior in response to environmental conditions and prey patchiness. A key prediction of foraging theory—that predator behavior should mirror the spatial and temporal distribution of prey (Stephens and Krebs 1987)—has been challenging to test in the wild because predator behavior and prey distribution are both, independently, influenced by multiple factors operating at different spatiotemporal scales. For example, large-scale climatic conditions can shape population-level dynamics in prey species and predict the distribution of predators at large scales (eg Pirotta et al. 2014; Barlow et al. 2020); however, the foraging behavior of predators is primarily determined by how prey behave at much finer spatiotemporal scales within their local environment (eg Torres et al. 2008). In other words, although the environment can predict prey dynamics at larger scales, this relationship may be less evident for predators at fine scales because they can only perceive the local environmental conditions (eg Cade et al. 2021). These local conditions are much more susceptible to noise and variation in terms of prey distribution and availability compared with what would be possible for predators to perceive if they had access to regional-scale information. In line with our first hypothesis, our results show that environmental conditions only partially predict prey availability at the fine spatiotemporal scale of the interaction site. We found that mullet schools—the main prey targeted by foraging dolphins and fishers—varied largely independently of most local environmental conditions, except for wind direction, which showed a clear effect. But why is that the case?

The large-scale dynamics driving mullet abundance at regional scales make mullet unpredictable to both dolphins and fishers foraging locally. During the austral autumn and winter, regional mullet availability increases along the coasts of northern Argentina and southern Brazil, as changes in sea water temperature and wind conditions trigger their reproductive migration (Sadowski and Almeida Dias 1986; Vieira and Scalabrin 1991). This pattern reflects a seasonal “resource wave” driven by spawning migration, with large mullet schools arriving along the coast within a narrow temporal window. Unlike some herring systems, it remains unclear whether this migration reflects discrete spawning populations. Regional temperature decline triggers migration, while local processes determine their accessibility in coastal and estuarine areas. Some predators can track environmental cues at this broader, regional scale, enabling them to follow such a resource wave. For example, the southern Brazilian industrial fishing fleet tracks water temperature conditions at regional scales to pursue migrating mullet schools (eg Lemos et al. 2016). However, the regional processes that bring mullet schools northward to spawn must align with local wind and current conditions to bring schools close to the coast, where they can enter estuaries and lagoons and become available to coastal resident dolphins (Teixeira et al. 2021) and artisanal fishers (Herbst and Hanazaki 2014).

Thus, different combinations of regional and local processes contribute to making mullet a resource that becomes available in time-limited waves. As illustrated by our fine-scale sonar data—in which mullet are primarily detected in large schools at irregular time intervals (Figs. S1 to S4)—mullet abundance at the scale perceived by dolphins and fishers is patchy and ephemeral. Local increases in mullet abundance are therefore likely to occur only when regional environmental processes (eg water temperature and wind conditions that influence the latitudinal migration of mullet schools) align with local conditions (eg wind direction and tidal currents that facilitate the movement of mullet schools in and out of estuaries). This multiscale structure may explain why dolphins do not necessarily forage more at the interaction site when mullets are regionally abundant. We found greater variation in the abundance of dolphins and fishers between days than within days. Such variation suggests either a response to broader-scale environmental variation or that some more competitive individual dolphins may maintain preferential access to this resource, potentially limiting access by conspecifics (Machado et al. 2024).

Contrary to our second hypothesis, predator abundance did not closely track local prey availability. We further found that most fine-scale environmental conditions were insufficient to predict either mullet or dolphin abundance. Mullet availability at the fine spatiotemporal scale of the interaction site was only partially influenced by wind direction—an environmental condition that operates across both local and the regional scales—and therefore reflects broader processes shaping mullet migratory dynamics (Lemos et al. 2016). This pattern suggests a mismatch between the spatial scale at which environmental conditions influence mullet migration and the fine-scale conditions experienced at the interaction site. Although declining water temperatures trigger mullet migration at regional scales, our results show that this signal alone does not predict local prey availability, which instead depends on additional local processes, such as wind and tidal conditions. Thus, mullet abundance at the interaction site may not be reliably inferred from local environmental cues alone, particularly given that mullet movements are driven by processes operating at broader spatial scales (Herbst and Hanazaki 2014; Lemos et al. 2016) and that fishers rely on dolphins as a primary cue for prey detection (Lang dos Santos et al. 2018). We did find that dolphins are more numerous during the flood phase of tidal currents, likely because these currents interact with the canal bathymetry, creating favorable foraging opportunities for dolphins (Pirotta et al. 2014). In Laguna, mullet schools entering the inlet typically move along the canal edges and seek refuge among the rocky jetties (Herbst and Hanazaki 2014). During flood tides, currents can dislodge mullets from these shelters, making them more susceptible to being herded by dolphins toward the line of fishers (Cantor et al. 2023). Thus, when regional conditions are suitable for mullet to approach the coast, they may become more available to dolphins, and thus to fishers, during flood tides (although flood tides during unsuitable regional conditions are unproductive).

The apparent unpredictability of mullet abundance at the interaction site reflects the difficulty of inferring local prey availability from environmental cues, rather than the absence of underlying environmental drivers, and has significant implications for the foraging tactics used by both artisanal fishers and wild dolphins. This pattern suggests that behavioral coordination may also improve the ability of predators to interpret noisy or unreliable local cues about prey availability. In this system, net-casting fishers employ a sit-and-wait foraging tactic at the interaction sites, relying almost entirely on dolphins to gain access to their prey, because synchronous social foraging produces nearly all successful catches. Dolphins, in turn, forage intensively in and around the nets when fish are captured (Cantor et al. 2023), suggesting that coordinated interactions may also provide foraging opportunities for dolphins, although this remains to be directly quantified. However, as in other multipredator systems, such interactions may also involve competitive components, particularly when prey availability is low, potentially constraining the net benefits of social foraging.

Consistent with our third hypothesis, behavioral synchrony is a key driver of foraging success for human fishers and is substantially more important than local mullet abundance in determining the success of this human–wildlife cooperation. This finding extends previous work showing that social foraging with dolphins increases fishers' catches (Simões-Lopes et al. 1998; Lang dos Santos et al. 2018) and builds on more recent evidence of synchronous interactions in this system (Cantor et al. 2023) by demonstrating that these benefits cannot be explained solely by the co-occurrence of predators during periods of high prey availability. Our results are also consistent with previous studies showing that artisanal fishers perceive clear benefits from cooperative interactions with dolphins, including increased catch success and reliability (Machado et al. 2019). Ultimately, our results highlight that behavioral coordination plays a central role in enabling predators to overcome the challenges posed by patchy and ephemeral prey.

## Supplementary Material

arag106_Supplementary_Data

## Data Availability

Analyses reported in this article can be reproduced using the data provided by Machado et al. (2026).

## Statement of inclusion

Our study brings together authors from diverse disciplinary backgrounds and career stages, including biologists and computer scientists from 4 countries. The leading authors were born, raised, trained, and are based in the country where the study was conducted. However, no author is an active participant in the artisanal fishing system studied. We acknowledge our position as external observers, shaped by our cultural backgrounds, privileges, and academic trajectories. To ensure a comprehensive and inclusive perspective, all authors contributed to the research and study design; this study is also part of a larger, long-term multidisciplinary effort that recognizes and respects the experiences and knowledge of local actors (artisanal fishers). We have made a concerted effort to cite literature from local scholars in the study region, including works published in the local language (Portuguese), to contextualize our findings within a broader ecological framework.
