# Supplementary material for: Synchronous human–dolphin foraging increases fishing success despite prey unpredictability
Source: Behav Ecol. 2026 Aug 28;37(6):arag106. doi: 10.1093/beheco/arag106 (PMC13596054; doi:10.1093/beheco/arag106)
Supplement: Supplementary file 1 [file arag106_supplementary_data.docx]

Supplementary material for the article:

Synchronous human-dolphin foraging increases fishing success despite prey unpredictability

**Table of contents**

[Are mullet schools patchily distributed and ephemeral?	2](#_Toc193668496)

[Testing the power law hypothesis for mullet detections	5](#_Toc193668497)

[Component Models	7](#_Toc193668498)

[Mullet	7](#_Toc193668499)

[Model coefficients	7](#_Toc193668500)

[Validation of model assumptions	7](#_Toc193668501)

[Dolphins	8](#_Toc193668502)

[Model coefficients	8](#_Toc193668503)

[Validation of model assumptions	8](#_Toc193668504)

[Fishers	9](#_Toc193668505)

[Model coefficients	9](#_Toc193668506)

[Validation of model assumptions	9](#_Toc193668507)

[Foraging Synchrony	10](#_Toc193668508)

[Model coefficients	10](#_Toc193668509)

[Validation of model assumptions	10](#_Toc193668510)

[Yield	11](#_Toc193668511)

[Model coefficients	11](#_Toc193668512)

[Validation of model assumptions	11](#_Toc193668513)

[References	12](#_Toc193668514)

# Are mullet schools patchily distributed and ephemeral?

We collected a total of 621,255 sonar frames in 117 sampling hours. After filtering off frames with predicted noise from the deep learning models, we kept 570,148 frames, of which 122,996 had at least one mullet detect (Figure S1). The distribution of mullet detections for all frames is shown in Figure S2, and the distribution by sampling day is shown in Figure S3.

 **Figure S1:** Mullet detections by sampling day. Blue shaded areas mark the duration of each sonar video, and each dot is a frame with mullet detection (frames with no mullet and/or with predicted noise were filtered off).

**Figure S2:** Distribution of mullet detections in 122,996 frames.

**Figure S3:** Distribution of mullet detections by sampling day. Frames with no mullet detections were filtered off.

### Testing the power law hypothesis for mullet detections

We tested if the distribution of mullet school sizes across noise-free frames across sampling hours followed a power law distribution. We used the poweRlaw R package (Gillespie, 2015) to fit a discrete power law distribution to the mullet detections frame-by-frame (Figure S4), filtering off frames with no mullet detections. We cannot reject the null hypothesis that the data is generated from a power law distribution (P = 0.379). This result for the frame-by-frame data reinforce the patchiness of mullet schools, as ca. 0.2% of the frames had mullet schools above the lower threshold of the power law distribution (277).

**Figure S4:** Results from 1,000 bootstraps using the frame-by-frame data of mullet detections. The first row shows the mean estimate of the lower threshold ($x_{\min}$), the scaling parameter ($\alpha$), the number of data points greater than or equal to the estimated lower threshold, and the p-value. The second row shows the estimate of standard deviation for each parameter. Black lines give the cumulative mean and standard deviation, and the red lines show the approximate 95% confidence intervals for each parameter using 90% of the bootstraps.

# Component Models

## Mullet

### Model coefficients

**Table S1:** Coefficients from the “Mullet” component model.

| **Predictor** | **Estimate** | **Std.Error** | **DF** | **Crit.Value** | **P.Value** | **Std.Estimate** |  |
| --- | --- | --- | --- | --- | --- | --- | --- |
| Tide velocity | -0.0389 | 0.0466 | 64 | -0.8359 | 0.4063 | -0.1000 |  |
| Wind direction | 0.6140 | 0.3037 | 64 | 2.0215 | 0.0474 | 0.3699 | * |
| Tidal currents | -0.0054 | 0.2097 | 64 | -0.0259 | 0.9794 | -0.0035 |  |
| Wind speed | -0.0077 | 0.0172 | 64 | -0.4501 | 0.6542 | -0.0676 |  |
| Water temperature | 0.0077 | 0.1058 | 64 | 0.0728 | 0.9422 | 0.0128 |  |

### Validation of model assumptions

**Figure S5:** Visual inspection of the homogeneity (a) and normality (b) of the residuals, and normality of the random effects (c) for the “Mullet” component model.

## Dolphins

### Model coefficients

**Table S2:** Coefficients from the “dolphins” component model.

| **Predictor** | **Estimate** | **Std.Error** | **DF** | **Crit.Value** | **P.Value** | **Std.Estimate** |  |
| --- | --- | --- | --- | --- | --- | --- | --- |
| Tide velocity | 0.0257 | 0.0252 | 65 | 1.0188 | 0.3121 | 0.1020 |  |
| Tidal currents | 0.2699 | 0.1112 | 65 | 2.4271 | 0.0180 | 0.2675 | * |
| Water temperature | -0.0059 | 0.0605 | 65 | -0.0980 | 0.9222 | -0.0152 |  |
| Mullet | 0.0585 | 0.0642 | 65 | 0.9119 | 0.3652 | 0.0904 |  |

### Validation of model assumptions

**Figure S6:** Visual inspection of the homogeneity (a) and normality (b) of the residuals, and normality of the random effects (c) for the “dolphins” component model.

## Fishers

### Model coefficients

**Table S3:** Coefficients from the “fishers” component model.

| **Predictor** | **Estimate** | **Std.Error** | **DF** | **Crit.Value** | **P.Value** | **Std.Estimate** |  |
| --- | --- | --- | --- | --- | --- | --- | --- |
| Dolphins | 0.1828 | 0.0865 | 63 | 2.1135 | 0.0385 | 0.1283 | * |
| Tide velocity | -0.0031 | 0.0210 | 63 | -0.1474 | 0.8833 | -0.0086 |  |
| Wind direction | 0.4724 | 0.2871 | 63 | 1.6453 | 0.1049 | 0.3084 |  |
| Tidal currents | 0.0492 | 0.1061 | 63 | 0.4637 | 0.6444 | 0.0342 |  |
| Wind speed | 0.0068 | 0.0102 | 63 | 0.6679 | 0.5066 | 0.0643 |  |
| Water temperature | 0.1330 | 0.0757 | 63 | 1.7571 | 0.0838 | 0.2396 |  |

### Validation of model assumptions

**Figure S7:** Visual inspection of the homogeneity (a) and normality (b) of the residuals, and normality of the random effects (c) for the “fishers” component model.

## Foraging Synchrony

### Model coefficients

**Table S4:** Coefficients from the “foraging synchrony” component model.

| **Predictor** | **Estimate** | **Std.Error** | **DF** | **Crit.Value** | **P.Value** | **Std.Estimate** |  |
| --- | --- | --- | --- | --- | --- | --- | --- |
| Dolphins | 0.5001 | 0.1819 | 67 | 2.749 | 0.0077 | 0.3063 | ** |
| Fishers | 0.3113 | 0.1392 | 67 | 2.236 | 0.0287 | 0.2716 | * |

### Validation of model assumptions

**Figure S8:** Visual inspection of the homogeneity (a) and normality (b) of the residuals, and normality of the random effects (c) for the “foraging synchrony” component model.

## Yield

### Model coefficients

**Table S5:** Coefficients from the “yield” component model.

| **Predictor** | **Estimate** | **Std.Error** | **DF** | **Crit.Value** | **P.Value** | **Std.Estimate** |  |
| --- | --- | --- | --- | --- | --- | --- | --- |
| Foraging synchrony | 0.8953 | 0.1961 | 67 | 4.566 | 0.000 | 0.4258 | *** |
| Mullet | 0.3887 | 0.1836 | 67 | 2.117 | 0.038 | 0.1748 | * |

### Validation of model assumptions

**Figure S9:** Visual inspection of the homogeneity (a) and normality (b) of the residuals, and normality of the random effects (c) for the “yield” component model.

# References

Gillespie, C. S. (2015). Fitting heavy tailed distributions: The poweRlaw package. *Journal of Statistical Software*, *64*(2), 1–16. <https://doi.org/10.18637/jss.v064.i02>
